# Risk for lung-related diseases associated with welding fumes in an occupational population: Evidence from a Cox model

**DOI:** 10.3389/fpubh.2022.990547

**Published:** 2022-08-25

**Authors:** Guangming Li, Jinfeng Jiang, Yonggang Liao, Siyu Wan, Yong Yao, Yongbin Luo, Xuyu Chen, Huiling Qian, Xiayun Dai, Wenjun Yin, Zhiteng Min, Guilin Yi, Xiaodong Tan

**Affiliations:** ^1^Department of Preventive Medicine, School of Public Health, Wuhan University, Wuhan, China; ^2^Wuhan Prevention and Treatment Center for Occupational Diseases, Wuhan, China; ^3^School of Health and Nurse, Wuchang University of Technology, Wuhan, China

**Keywords:** welding fumes, occupational population, lung-related diseases, Cox model, risk

## Abstract

**Background:**

Welding fumes are a risk factor for welder pneumoconiosis. However, there is a lack of population information on the occurrence of welding fume-induced lung cancer, and little is known about the welding fume pathogenesis.

**Methods:**

Welding fume and metal ion concentrations were assessed in a vehicle factory in Wuhan. A Cox regression model estimated lung-related disease risk in workers by independent and combined factors.

**Results:**

Workers' exposures were divided into four grades; the highest exposure was among the welders in the maintenance workshop, the highest Mn and Fe exposure was 4 grades, and the highest Cr exposure was 3 grades. Subgroup analysis found that the risk of lung-related disease was 2.17 *(95% CI: 1.31–3.57, p *<* 0.05)* in welders compared with non-welders, and the risk of pulmonary disease in male welders was 2.24 *(95% CI: 1.34–3.73, p *<* 0.05)* compared to non-welders. Smoking welders had a 2.44 *(95% CI: 1.32–4.51, p *<* 0.01)* higher incidence of lung-related diseases than non-welders. Total years of work as an independent protective factor for lung-related disease risk was 0.72 *(95% CI: 0.66–0.78, p *<* 0.01)*. As an independent risk factor, high-high and high-low exposure had a 5.39 *(95% CI: 2.52–11.52, p *<* 0.001)* and 2.17 *(95% CI: 1.07–4.41, p *<* 0.05)* higher risk for lung-related diseases, respectively.

**Conclusions:**

High welding fume exposure is a significant risk factor for lung-related disease in workers.

## Introduction

In 2010, the International Labor Organization estimated that there were 3.5 billion economically active people and possibly 11 million welders in the world (1). Figures published by the World Health Organization show that welders make up an average of 0.31% of the economically active population (2). A total of 746.52 million individuals were employed in China in 2021, and the preliminary estimate is that there are 2.3 million welders in China (3). According to the World Health Organization, the number of people exposed to welding fumes may be 10 times higher than the number of people with welder titles. This suggests that the number of people exposed to welding fumes in China may be close to 23 million workers, or 3.1% of the country's economically active population. Welding fumes were classified as Class 2B carcinogens by the World Health Organization's International Agency for Research on cancer in 1989 and upgraded to Class I carcinogens in 2017 (4, 5), and possible carcinogenic mechanisms were published online in 2018.

Welding fume exposure has become an urgent concern in the field of occupational health. For example, the Swedish working environment authority reported an estimate of 71 deaths per year in Sweden (based on 2016 data) that may be directly related to welding fumes (6). In addition, a UK study found that an estimated 152 people die each year from occupational exposure to welding fumes (lung cancer only) (7). Due to the large amount of toxic and harmful substances, such as welding fumes, metal oxide particles, ozone, nitrogen oxide, and CO, the whole workshop will usually be filled with small particles of hazardous substances (8). Welders who lack protective equipment are exposed to potentially dangerous welding fumes for long periods of time. In welding fume simulation tests, animal experimental studies and a small number of population retrospective surveys, the health effects of welding fumes were found to include effects on the respiratory, neurological, eye and skin, renal, immune, reproductive, cardiovascular and liver systems;, genetic chromosomes; and lipid peroxidation (9–11). However, due to the lack of large population-based cohort studies, studies on the pathogenesis of welding fumes in the population are still scarce.

Although China's industry is developing rapidly, research on welder's health in China is relatively behind, and the research contents, depth and scope obviously lag behind those of developed Western countries; addressing this lack of research is important to further understand the impacts on Chinese welder health. The purpose of this study was to investigate the health status of welders in a vehicle factory in Wuhan and to construct a retrospective cohort study to explore the influencing factors on the occurrence of lung-related diseases among welders, providing support for the occupational health protection of future welding workers.

## Materials and methods

### Study setting

This study was conducted in a vehicle factory in Wuhan. With the rapid development of the Chinese economy, the vehicle factory in Wuhan has undergone changes and several site reconstructions, but the manufacturing process and processes of the vehicle factory have not changed much, and the types of welding rods and wires used have not changed significantly. Although the annual consumption of welding material and steel in the plant has increased annually, it has not fluctuated much, indicating a stable working environment and fixed jobs.

### Data collection

#### Workshop dust and metal content monitoring

According to the sampling standard (GBZ 159−2004) (12) of workplace air hazardous substance monitoring and the actual situation of site investigation, sampling points were set up. Individual sampling was performed at the height of the individual's breathing belt, and dust and metals in the air were collected for at least 120 min using polyvinyl chloride and cellulose acetate filters at 1 L/min using Gilair Plus. Fixed-point sampling was conducted using an FCC-30 (Jiangsu Yancheng Tianyue Instrument Co., Ltd.) two-head dust and metal sampler at the individual respiratory belt height, and dust and metal contents in the air were collected using polyvinyl chloride and cellulose acetate filters. The air flow was sampled at 20 L/min for 15 min. The dust quality was measured by a ppm balance, and the metal content was detected by inductively coupled plasma—optical emission spectrometry (ICP–OES) (Perkineimer Avio200) (13, 14). According to the occupational exposure level of harmful chemical factors and its classification and control method, the exposure level was divided into 5 levels: level 0 [≤ 1% occupational exposure limits (OEL)] was basically no exposure, level 1 (>1%, ≤ 10% OEL) was extremely low and had no correlation effect, grade 2 (>10%, ≤ 50% OEL) had exposure but no significant health effects, grade 3 (>50%, ≤ OEL) had significant exposure and required action to restrict activity, and grade 4 (>OEL) exceeded OELs, with a higher grade representing greater health hazards after exposure. According to the concentration of exposure and the level of exposure, the risk level of exposure was divided into high-high exposure, high-low exposure, low-high exposure, and low-low exposure according to the part and type of work.

#### Survey of subjects and physical examination

According to the requirement of physical examination for occupational diseases, we collected data *via* clinical interviews, including general demographic characteristics, disease history, occupational history and personal life behavior. In addition, lung ventilation, electrocardiography, chest X-ray, blood pressure, height, weight, and waist circumference were measured. For height and weight measurements, participants were reminded to remove their shoes and heavy clothing, and repeated measurements were averaged to the nearest 0.1 cm and 0.1 kg, respectively. The waist circumference was measured at the level of the anterior superior iliac crest and the midpoint of the inferior edge of the 12th rib for 1 week, and the reading was accurate to 0.1 cm. Hypertension was defined as systolic blood pressure (SBP) ≥140 mm Hg and/or diastolic blood pressure (DBP) ≥90 mm Hg, previous diagnosis of hypertension, or use of antihypertensive medication. Forced vital capacity in one second (FEV1)/ forced vital capacity (FVC) < 0.7 was the criterion for pulmonary ventilation impairment, and diagnostic criteria such as restrictive pulmonary ventilation impairment referred to spirometry guidelines (15). Definition of sleep status, good (Lie down to sleep), normal (Fall asleep quickly and occasionally dream), bad (Difficulty falling asleep in bed and frequent nightmares), and very bad (Need sleeping pills to help sleep). BMI groups were low, middle, and high (body mass index, strata of <18.5, 18.5–24, >24 kg/m^2^). Total working years refers to work from the time one left school, not specifically work in a research plant.

### Exposure control

In this study, we constructed an exposure-time response relationship based on the time of diagnosis of pulmonary function tests, chest X-rays, and lung-related diseases (bronchitis, asthma, emphysema, pleurisy, chronic obstructive pulmonary disease, pneumoconiosis, and other lung diseases), constructed the time from exposure to diagnosis of lung related diseases, and excluded those who had lung-related diseases before exposure. Cox proportional hazards model, as a semiparametric regression model, can explore one or more variables on the impact of disease risk factors. Therefore, the cox proportional hazards model was used to explore age, sex, education, exposure level etc. of lung-related diseases during the exposure period.

### Statistical analysis

Continuous variables are expressed as the mean and standard deviation, and discrete variables are expressed as the median and interquartile range. Normally distributed data were analyzed by the pairwise comparison *t*-test, and non-normally distributed data were assessed by the chi-square test or non-parametric Wilcoxon signed rank sum test. All statistical tests were two-sided, and a *p*-value < 0.05 was considered statistically significant. Data analyses were run in R version 3.6.2 (R Foundation for Statistical Computing, Vienna, Austria). We used the function “cox.zph” of the “survival” package to test the proportional hazards assumption.

## Results

### Level of environmental monitoring and exposure

The total concentration of welding fumes was 0.467 mg/m^3^, and no metal ion manganese was detected (Table 1). The concentration of welding fumes reached level 2, and the concentration of iron was 0.00284 mg/m^3^, reaching level 1. The exposure level of Mn, Cr and Ni was 0. The individual welding fume exposure of the non-electric welder in the manufacturing workshop was 0.892 mg/m^3^, and the fixed-point welding fume exposure was 2.083 mg/m^3^, reaching levels 2 and 3, respectively. The individual Cr concentration was 0.000603 mg/m^3^, the fixed-point Cr concentration was 0.000552 mg/m^3^, the fixed-point Cr concentration was 0.000603 mg/m^3^, the fixed-point Cr concentration was 0.000603 mg/m^3^, and the fixed-point Cr concentration was 0.000552 mg/m^3^; both reached level 1 exposure. In the manufacturing workshop, the individual welding fume exposure was 10.073 mg/m^3^, the fixed-point welding fume exposure was 1.133 mg/m^3^, the individual Cr exposure concentration was 0.0141 mg/m^3^, and the fixed-point Cr exposure concentration was 0.000542 mg/m^3^, which reached grades 3 and 1, respectively. The blank control welding fume level in the assembly workshop was 1.7 mg/m^3^, reaching level 2, and the iron concentration was 0.0608 mg/m^3^, reaching the contact level of grade 1. The concentrations of Mn, Cr, and Ni were lower, all reaching the contact level of 0. The individual welding fume exposure of non-welders in the maintenance workshop was 9.619 mg/m^3^, and the fixed spot welding fume level was 2.483 mg/m^3^, which reached levels 4 and 3, respectively. The exposure concentration of Cr reached levels of 2 and 0, respectively, and the exposure concentration of Mn reached levels 3 and 1, respectively. The welding fume exposure of the individual welder in the maintenance workshop was 39.612 mg/m^3^, and the fixed spot welding fume exposure was 2.6 mg/m^3^, which reached levels 4 and 3, respectively. The exposure concentration of Cr reached levels 3 and 0, and the exposure concentration of Mn reached levels 4 and 1, respectively. In the maintenance workshop, the blank control welding fume level was 1.967 mg/m^3^, reaching the contact level of 2, the iron concentration was 0.0719 mg/m^3^, reaching the contact level of 2, the concentration of Mn, Cr, and Ni was higher, reaching a contact level of 0.0719 mg/m^3^, with exposure levels of 1, 1, and 0, respectively.

**Table 1:** Concentration and contact grade of welding fume and metal ions in air.

		**Mean**	**P50**	**P25**	**P75**	**Min**	**Max**	**PC-TWA (40h)**	**PC-TWA (72h)**	**PC-TWA (Maximum working hours)**	**Risk Level**
Weld fume	Non-welder of manufacturing workshop	0.891674	1.015453	0.518018	1.141553	0.518018	1.141553	4	1.668	1.056	2
	Welder of manufacturing workshop	10.07298	3.430657	2.556391	24.23188	2.556391	24.23188	4	1.668	1.056	4
	Fixed point non-welder of manufacturing workshop	2.083333	2.083333	1.833333	2.333333	1.833333	2.333333	4	1.668	1.056	3
	Fixed point welder of manufacturing workshop	1.133333	1.133333	0.633333	1.633333	0.633333	1.633333	4	1.668	1.056	2
	Blank of manufacturing workshop	1.7	1.7	1	2.4	1	2.4	4	1.668	1.056	2
	Fixed point average of manufacturing workshop	3.25	3.533333	2.55	3.95	1.666667	4.266667	4	1.668	1.056	3
	Non-welder of repair workshop	9.61907	9.61907	9.372549	9.865591	9.372549	9.865591	4	1.668	1.056	4
	Welder of repair workshop	39.61187	39.61187	11.3242	67.89954	11.3242	67.89954	4	1.668	1.056	4
	Fixed point non-welder of repair workshop	2.483333	2.483333	1.866667	3.1	1.866667	3.1	4	1.668	1.056	3
	Fixed point welder of repair workshop	2.6	2.6	2.433333	2.766667	2.433333	2.766667	4	1.668	1.056	3
	Blank of repair workshop	1.966667	1.966667	1.233333	2.7	1.233333	2.7	4	1.668	1.056	2
	Fixed point average of repair workshop	4.316667	4.316667	2.6	6.033333	2.6	6.033333	4	1.668	1.056	4
	Factory environment blank	0.466667	0.466667	0.466667	0.466667	0.466667	0.466667	4	1.668	1.056	2
Cr	Non-welder of manufacturing workshop	0.000603	0.000424	0.00031	0.001075	0.00031	0.001075	0.05	0.02085	0.0132	1
	Welder of manufacturing workshop	0.014135	0.018539	0.002283	0.021583	0.002283	0.021583	0.05	0.02085	0.0132	3
	Fixed point non-welder of manufacturing workshop	0.000552	0.000552	0.000372	0.000731	0.000372	0.000731	0.05	0.02085	0.0132	1
	Fixed point welder of manufacturing workshop	0.000542	0.000542	0.000125	0.000959	0.000125	0.000959	0.05	0.02085	0.0132	1
	Blank of manufacturing workshop	0.000375	0.000376	0.000179	0.000572	0.000179	0.000572	0.05	0.02085	0.0132	0
	Fixed point average of manufacturing workshop	0.000801	0.000902	0.000417	0.001083	0.000417	0.001083	0.05	0.02085	0.0132	1
	Non-welder of repair workshop	0.007699	0.007699	0.005342	0.010055	0.005342	0.010055	0.05	0.02085	0.0132	2
	Welder of repair workshop	0.019987	0.019987	0.00578	0.034193	0.00578	0.034193	0.05	0.02085	0.0132	3
	Fixed point non-welder of repair workshop	0.00029	0.00029	0.00016	0.000419	0.00016	0.000419	0.05	0.02085	0.0132	0
	Fixed point welder of repair workshop	0.000332	0.000332	0.000052	0.000612	0.000052	0.000612	0.05	0.02085	0.0132	0
	Blank of repair workshop	0.000732	0.000732	0.000157	0.001307	0.000157	0.001307	0.05	0.02085	0.0132	1
	Fixed point average of repair workshop	0.000657	0.000657	0.000249	0.001064	0.000249	0.001064	0.05	0.02085	0.0132	1
	Factory environment blank	0.00002	0.00002	0.000002	0.000038	0.000002	0.000038	0.05	0.02085	0.0132	0
Fe	Non-welder of manufacturing workshop	0.091064	0.061466	0.057313	0.154414	0.057313	0.154414	0.25	0.10425	0.066	2
	Welder of manufacturing workshop	2.158942	2.848741	0.443535	3.18455	0.443535	3.18455	0.25	0.10425	0.066	4
	Fixed point non-welder of manufacturing workshop	0.077827	0.077827	0.053892	0.101762	0.053892	0.101762	0.25	0.10425	0.066	1
	Fixed point welder of manufacturing workshop	0.080742	0.080742	0.023315	0.13817	0.023315	0.13817	0.25	0.10425	0.066	1
	Blank of manufacturing workshop	0.060833	0.060833	0.025013	0.096653	0.025013	0.096653	0.25	0.10425	0.066	1
	Fixed point average of manufacturing workshop	0.11008	0.129121	0.055813	0.145305	0.055813	0.145305	0.25	0.10425	0.066	2
	Non-welder of repair workshop	1.262596	1.262596	0.871609	1.653583	0.871609	1.653583	0.25	0.10425	0.066	4
	Welder of repair workshop	3.792913	3.792913	1.305619	6.280207	1.305619	6.280207	0.25	0.10425	0.066	4
	Fixed point non-welder of repair workshop	0.07114	0.07114	0.045448	0.096832	0.045448	0.096832	0.25	0.10425	0.066	2
	Fixed point welder of repair workshop	0.068534	0.068534	0.012406	0.124663	0.012406	0.124663	0.25	0.10425	0.066	2
	Blank of repair workshop	0.071886	0.071886	0.033234	0.110538	0.033234	0.110538	0.25	0.10425	0.066	2
	Fixed point average of repair workshop	0.105586	0.105586	0.081386	0.129786	0.081386	0.129786	0.25	0.10425	0.066	2
	Factory environment blank	0.002839	0.002839	0.002357	0.00332	0.002357	0.00332	0.25	0.10425	0.066	1
Mn	Non-welder of manufacturing workshop	0.010721	0.00781	0.004992	0.019362	0.004992	0.019362	0.15	0.06255	0.0396	1
	Welder of manufacturing workshop	0.282111	0.352849	0.039982	0.453504	0.039982	0.453504	0.15	0.06255	0.0396	4
	Fixed point non-welder of manufacturing workshop	0.010131	0.010131	0.006542	0.013719	0.006542	0.013719	0.15	0.06255	0.0396	1
	Fixed point welder of manufacturing workshop	0.010261	0.010261	0.002809	0.017713	0.002809	0.017713	0.15	0.06255	0.0396	1
	Blank of manufacturing workshop	0.007001	0.007001	0.002813	0.01119	0.002813	0.01119	0.15	0.06255	0.0396	0
	Fixed point average of manufacturing workshop	0.014336	0.016768	0.004837	0.021405	0.004837	0.021405	0.15	0.06255	0.0396	1
	Non-welder of repair workshop	0.124932	0.124932	0.094887	0.154976	0.094887	0.154976	0.15	0.06255	0.0396	3
	Welder of repair workshop	0.34695	0.34695	0.092056	0.601845	0.092056	0.601845	0.15	0.06255	0.0396	4
	Fixed point non-welder of repair workshop	0.003985	0.003985	0.002223	0.005747	0.002223	0.005747	0.15	0.06255	0.0396	1
	Fixed point welder of repair workshop	0.005633	0.005633	0.00064	0.010626	0.00064	0.010626	0.15	0.06255	0.0396	1
	Blank of repair workshop	0.002909	0.002909	0.000855	0.004963	0.000855	0.004963	0.15	0.06255	0.0396	1
	Fixed point average of repair workshop	0.005614	0.005614	0.002327	0.008901	0.002327	0.008901	0.15	0.06255	0.0396	1
	Factory environment blank							0.15	0.06255	0.0396	0
Ni	Non-welder of manufacturing workshop	0.000299	0.000186	0.00017	0.000542	0.00017	0.000542	1	0.417	0.264	0
	Welder of manufacturing workshop	0.006827	0.008936	0.002015	0.009531	0.002015	0.009531	1	0.417	0.264	0
	Fixed point non-welder of manufacturing workshop	0.000389	0.000389	0.000355	0.000423	0.000355	0.000423	1	0.417	0.264	0
	Fixed point welder of manufacturing workshop	0.000329	0.000329	0.000134	0.000524	0.000134	0.000524	1	0.417	0.264	0
	Blank of manufacturing workshop	0.000192	0.000192	0.000114	0.00027	0.000114	0.00027	1	0.417	0.264	0
	Fixed point average of manufacturing workshop	0.000379	0.000398	0.000306	0.000433	0.000306	0.000433	1	0.417	0.264	0
	Non-welder of repair workshop	0.004897	0.004897	0.004321	0.005474	0.004321	0.005474	1	0.417	0.264	0
	Welder of repair workshop	0.011883	0.011883	0.007891	0.015875	0.007891	0.015875	1	0.417	0.264	1
	Fixed point non-welder of repair workshop	0.000532	0.000532	0.000159	0.000906	0.000159	0.000906	1	0.417	0.264	0
	Fixed point welder of repair workshop	0.000279	0.000279	0.000046	0.000512	0.000046	0.000512	1	0.417	0.264	0
	Blank of repair workshop	0.000211	0.000211	0.000142	0.00028	0.000142	0.00028	1	0.417	0.264	0
	Fixed point average of repair workshop	0.000362	0.000362	0.000236	0.000488	0.000236	0.000488	1	0.417	0.264	0
	Factory environment blank	0.000035	0.000035	0.00002	0.00005	0.00002	0.00005	1	0.417	0.264	0

The table shows only the main metal elements in welding fumes and the allowable exposure limits for the working hours per week.

### Data description

A total of 427 diagnostic datas were collected; 13 invalid diagnostic datas were excluded, and 414 valid datas were obtained. Twenty-nine diagnostic datas were missing after excluding ECG, pulmonary ventilation function test and clinical medical examination, and data from a total of 384 individuals were obtained, with an effective rate of 89.93%. Among them, welding workers and non-welding workers accounted for 50% each. In comparison with non-welders, there were significant differences in sex, 3-month neurological symptoms, 3-month lung symptoms, working posture, daily working hours, lung-related diseases, FEV1, exposure level, and the use of a mask and welding face screen (*p* < 0.05) (Table 2).

**Table 2:** General information description for welders and non-welders.

	**Non-welder** ***N = 192***	**Welder** ***N = 192***	***p***
**Sex**			0.002
Man	188 (97.9%)	172 (89.6%)	
Female	4 (2.08%)	20 (10.4%)	
**BMI**			0.279
Low (18.5 < BMI)	7 (3.65%)	5 (2.60%)	
Middle (18.5 ≤ BMI ≤ 24)	71 (37.0%)	86 (44.8%)	
High (BMI>24)	114 (59.4%)	101 (52.6%)	
**Abdominal obesity**			0.443
No	127 (66.1%)	135 (70.3%)	
Yes	65 (33.9%)	57 (29.7%)	
**Marriage:**			0.135
Unmarried	31 (16.1%)	35 (18.2%)	
Married	160 (83.3%)	151 (78.6%)	
Other	1 (0.52%)	6 (3.12%)	
**Education:**			0.313
Low level	44 (22.9%)	35 (18.2%)	
High level	148 (77.1%)	157 (81.8%)	
**Disease history**			0.306
No	109 (56.8%)	98 (51.0%)	
Yes	83 (43.2%)	94 (49.0%)	
**Hypertension**			0.820
No	140 (72.9%)	137 (71.4%)	
Yes	52 (27.1%)	55 (28.6%)	
**Neurological related symptoms for 3 months:**			0.019
No	111 (57.8%)	87 (45.3%)	
Yes	81 (42.2%)	105 (54.7%)	
**Lung related symptoms for 3 months**			<0.001
No	173 (90.1%)	134 (69.8%)	
Yes	19 (9.90%)	58 (30.2%)	
**Eye related symptoms for 3 months**			0.757
No	107 (55.7%)	111 (57.8%)	
Yes	85 (44.3%)	81 (42.2%)	
**Working posture**			<0.001
Curl up	6 (3.12%)	4 (2.08%)	
Squat	42 (21.9%)	90 (46.9%)	
Station	133 (69.3%)	90 (46.9%)	
Other	11 (5.73%)	8 (4.17%)	
**Whether to work shift**			0.072
No	151 (78.6%)	137 (71.4%)	
Two shifts	40 (20.8%)	49 (25.5%)	
Three shifts	1 (0.52%)	6 (3.12%)	
**Smoking**			0.079
No	58 (30.2%)	79 (41.1%)	
Yes	127 (66.1%)	108 (56.2%)	
Quit	7 (3.65%)	5 (2.60%)	
**Drinking**			0.147
No	112 (58.3%)	129 (67.2%)	
Yes	78 (40.6%)	60 (31.2%)	
Quit	2 (1.04%)	3 (1.56%)	
**Exercise within half a year**			0.727
No	52 (27.1%)	48 (25.0%)	
Yes	140 (72.9%)	144 (75.0%)	
**Sleep state**			0.915
Good	67 (34.9%)	71 (37.0%)	
Normal	102 (53.1%)	98 (51.0%)	
Bad	23 (12.0%)	22 (11.5%)	
Very bad	0 (0.00%)	1 (0.52%)	
**Often take a nap**			0.424
No	143 (74.5%)	135 (70.3%)	
Yes	49 (25.5%)	57 (29.7%)	
**Lung related disease**			0.042
No	167 (87.0%)	151 (78.6%)	
Yes	25 (13.0%)	41 (21.4%)	
**ECG**			1.000
No	158 (82.3%)	159 (82.8%)	
Yes	34 (17.7%)	33 (17.2%)	
**Exposure level**			<0.001
Low_low	103 (53.6%)	5 (2.60%)	
Low_high	75 (39.1%)	3 (1.56%)	
High_low	7 (3.65%)	123 (64.1%)	
High_high	7 (3.65%)	61 (31.8%)	
**Mask**			<0.001
No	7 (3.65%)	1 (0.52%)	
Dust prevent	163 (84.9%)	187 (97.4%)	
Medical	16 (8.33%)	4 (2.08%)	
Poison	6 (3.12%)	0 (0.00%)	
**Welding surface screen**			<0.001
No	61 (31.8%)	14 (7.29%)	
Yes	11 (5.73%)	169 (88.0%)	
Protective goggles	120 (62.5%)	9 (4.69%)	
**Ear protection**			0.455
No	95 (49.5%)	95 (49.5%)	
Both earplugs and earflap	22 (11.5%)	30 (15.6%)	
Earplugs	74 (38.5%)	67 (34.9%)	
Earflap	1 (0.52%)	0 (0.00%)	
Age [M (P25, P75), year]	46 (38.75, 55)	43 (36.75, 54)	0.013
Workday/week [M (P25, P75), days]	6 (6, 6)	6 (6, 6)	0.08
Worktime/day [M (P25, P75), h]	8 (8, 8)	8 (8, 10)	0.006
Total working years [M (P25, P75), year]	20 (12, 26)	17 (10, 24.2)	0.03
FVC(L)	3.76 (0.62)	3.64 (0.66)	0.074
FEV1(L)	3.24 (0.53)	3.13 (0.55)	0.047
FEV1/FVC% (%) [M (P25, P75)]	86.3 (82.8, 89.6)	85.6 (81.9, 89.9)	0.6
FVC% (%) [M (P25, P75)]	95.5 (87.0, 104.9)	95.6 (86.6, 103.7)	0.7
FEV1% (%) [M (P25, P75)]	92.8 (85.6, 100.3)	91.5 (85.3, 97.8)	0.2

ECG, Electrocardiography; FVC, forced vital capacity; FEV1, Forced vital capacity in 1 s; FVC%, FVC as a percentage of predicted value; FEV1%, FEV1 as a percentage of predicted value.

### Subgroup analyses

**Figure 1:**
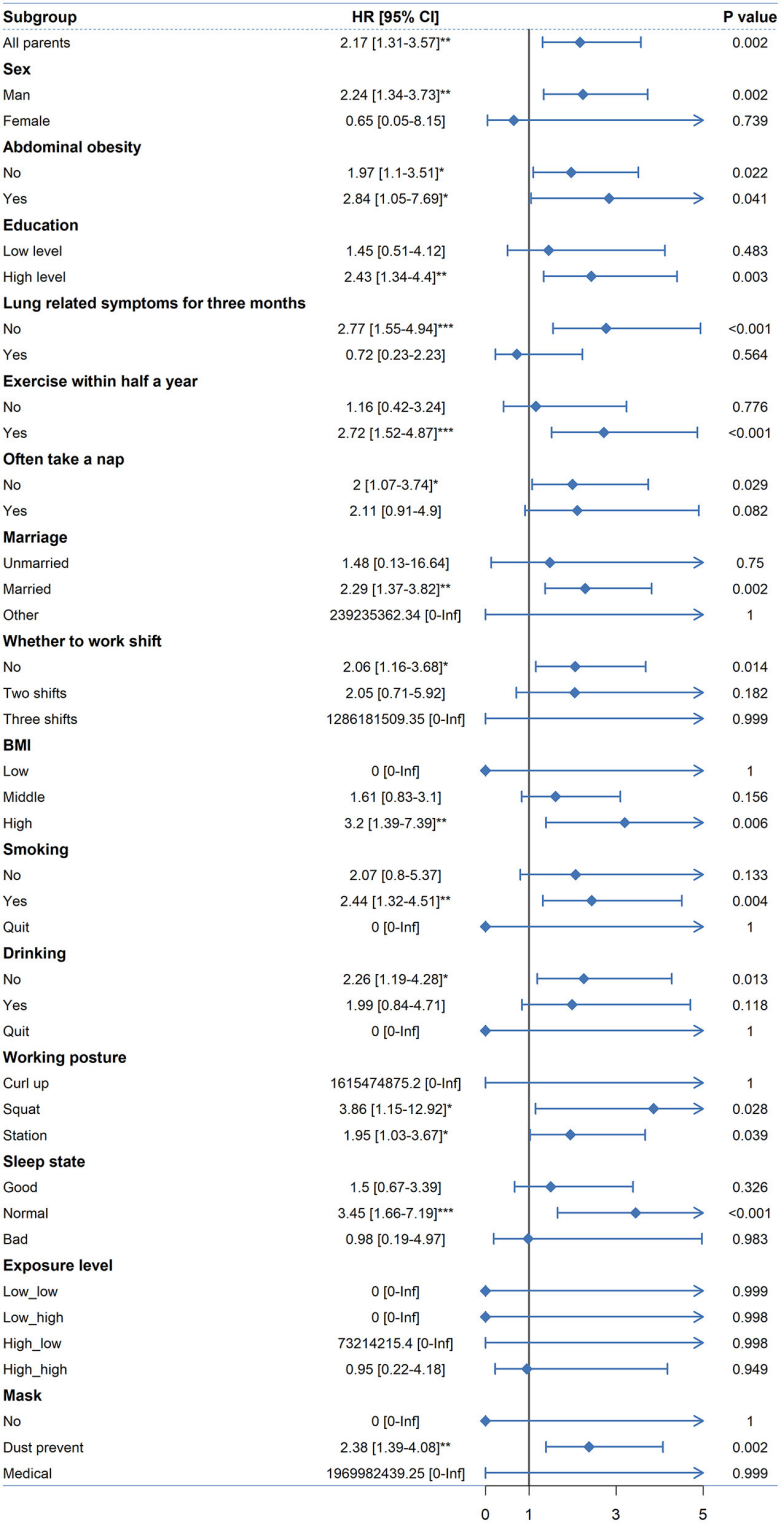
Cox model subgroup analysis of lung-related diseases and exposure to welding fumes among welders and non-welders. (*p < 0.05; **p < 0.01; ***p < 0.001).

Figure 1 summarizes the subgroup-specific hazard ratio (HR) estimates between lung-related disease and welder status. The risk of lung-related disease among welders vs. non-welders in the overall population was 2.17 *(95% CI: 1.31–3.57, p *<* 0.05)*, the risk of pulmonary disease in male welders was 2.24 *(95% CI: 1.34–3.73, p *<* 0.05)* in non-welders, and welders with abdominal obesity were 2.84 *(95% CI: 1.05–7.69, p *<* 0.05)* times more likely to develop lung-related diseases than welders without abdominal obesity who were at a risk of 1.97 *(95% CI: 1.1–3.51, p *<* 0.05)*. The risk of lung-related disease among welders with a high level of education was 2.43 *(95% CI: 1.34–4.4, p *<* 0.01)* compared with non-welders. Among those who did not have lung-related symptoms within 3 months, welders were 2.77 *(95% CI: 1.55–4.94, p *<* 0.001)* times more likely to develop lung-related disease than non-welders. The risk among welders who exercised within 6 months was 2.72 *(95% CI: 1.52–4.87, p *<* 0.001)*. The incidence of lung-related diseases was 2 *(95% CI: 1.07–3.74, p *<* 0.05)* in the welders who did not nap frequently. Married welders had a 2.29 *(95% CI: 1.37–3.82, p *<* 0.01)* higher incidence of lung-related diseases than non-welders. Non-shift welders were 2.06 *(95% CI: 1.16–3.68, p *<* 0.05)* more likely to develop lung-related diseases than non-shift welders. Smoking welders had a 2.44 *(95% CI: 1.32–4.51, p *<* 0.01)* higher incidence of lung-related diseases than non-welders. The incidence of lung-related diseases in non-alcoholic welders was 2.26 *(95% CI: 1.19–4.28, p *<* 0.05)*. The incidence of lung-related diseases was 3.86 *(95% CI: 1.15–12.92, p *<* 0.05)*. The welders with normal sleep quality were 3.45 *(95% CI: 1.66–7.19, p *<* 0.001)* more likely to develop lung-related disease than non-welders. The prevalence of lung-related diseases was 2.38 *(95% CI: 1.39–4.08, p *<* 0.001)* in non-welders.

### Independent risk factors for lung-related diseases

**Figure 2:**
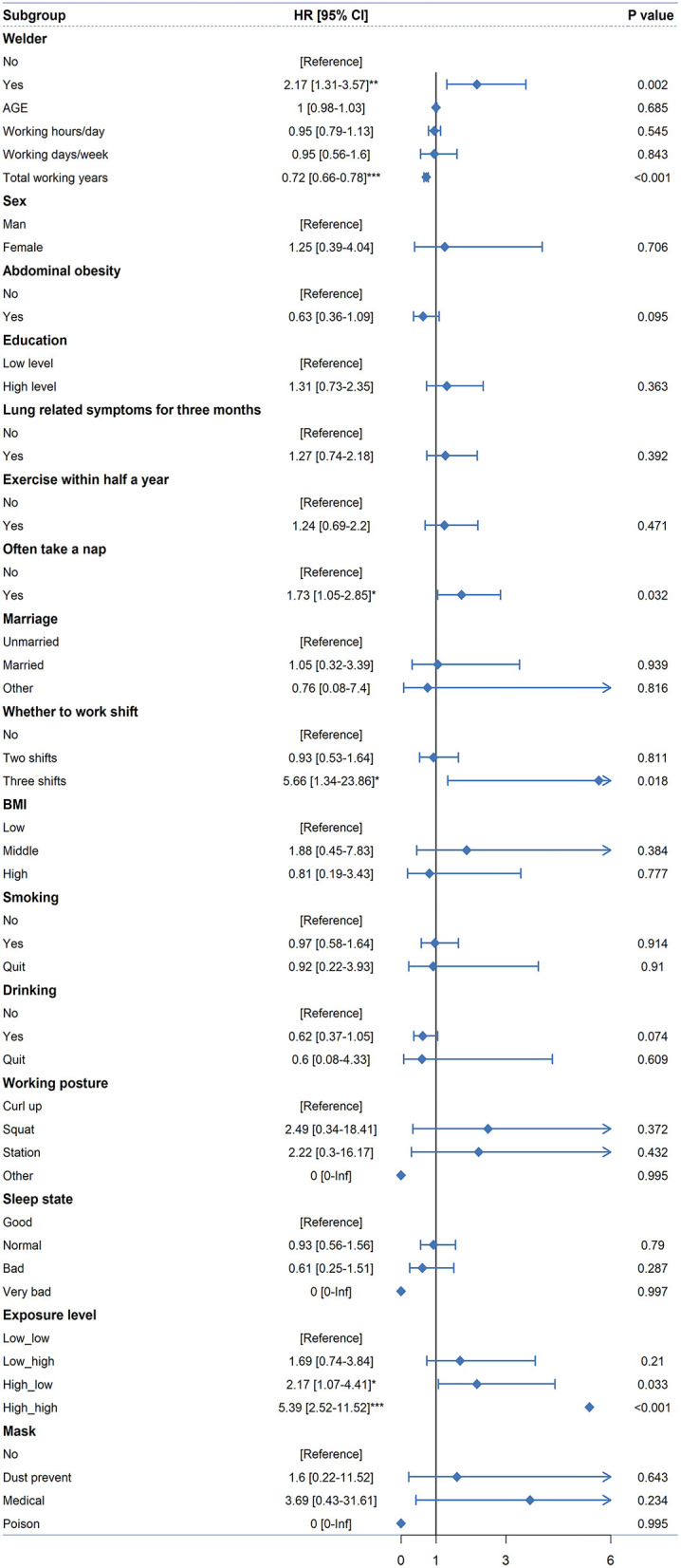
Independent risk factors for lung-related diseases. (*p < 0.05; **p < 0.01; ***p < 0.001).

Figure 2 summarizes the independent risk factors for lung-related diseases. Being a welder, total working years, frequent napping, shift work and exposure to welding fumes were independent risk factors for lung-related diseases. For example, the risk of developing lung-related disease decreased by a factor of 0.72 *(95% CI: 0.66–0.78, p *<* 0.01)* with increasing total years of work. The increases in the risk for lung-related diseases were 5.39 *(95% CI: 2.52–11.52, p *<* 0.001)* and 2.17 *(95% CI: 1.07–4.41, p *<* 0.05)* for high-high and high-low exposure compared to low-low exposure, respectively.

### Combined factors affecting lung-related diseases

Independent influencing factors were included in the multivariate Cox regression analysis, fulfilling the PH assumption (Supplementary Table 1). It was found that total working years and exposure levels were the joint risk factors for the occurrence of lung-related diseases after controlling whether the welders took lunch breaks frequently and whether they were on shift (Table 3). For example, an increase in total years of work was protective against the development of lung-related disease by a factor of 0.69 *(95% CI: 0.63–0.756, p *<* 0.001)*, and the risk factors for lung-related diseases were 2.80 *(95% CI: 1.14–6.87, p *<* 0.05)* and 4.14 *(95% CI: 1.25–13.70, p *<* 0.05)* that low_high and high_high exposure level respectively than low_low exposure level.

**Table 3:** Multivariate Cox model and sensitivity analysis model of influences lung-related diseases.

	**Multivariate COX**		**Model 1**		**Model 2**		**Model 3**	
	**HR (95% CI)**	***P***	**HR (95% CI)**	***P***	**HR (95% CI)**	***P***	**HR (95% CI)**	**P**
Welder							
No					(Reference)		(Reference)	
Yes	1.48 (0.54–4.04)	0.44			1.41 (0.51–3.95)	0.51	1.42 (0.53–3.82)	0.48
Total working years	0.69 (0.63–0.76)	0.00	0.69 (0.63–0.76)	*p < * 0.001	0.69 (0.63–0.76)	*p < * 0.001	0.69 (0.63–0.76)	*p < * 0.001
Often take a nap							
No			(Reference)				(Reference)	
Yes	1.68 (0.99–2.83)	0.05	1.65 (0.981–2.79)	0.06			1.72 (1.03–2.90)	0.04
Whether to work shift							
No			(Reference)		(Reference)			
Two shifts	1.32 (0.70–2.50)	0.40	1.28 (0.68–2.43)	0.45	1.43 (0.75–2.72)	0.27		
Three shifts	3.26 (0.70–15.15)	0.13	3.29 (0.71–15.29)	0.13	3.35 (0.72–15.53)	0.12		
Exposure level							
Low_low			(Reference)		(Reference)		(Reference)	
Low_high	2.80 (1.14–6.87)	0.02	2.80 (1.14–6.86)	0.03	3.25 (1.34–7.87)	*p < * 0.01	2.53 (1.06–6.04)	0.04
High_low	1.20 (0.37–3.86)	0.77	1.71 (0.82–3.56)	0.15	1.39 (0.43–4.54)	0.5	1.29 (0.41–4.09)	0.67
High_high	4.14 (1.25–13.70)	0.02	5.80 (2.55–13.20)	*p < * 0.001	4.88 (1.48–16.11)	*p < * 0.01	3.91 (1.20–12.70)	0.02

### Sensitivity analyses

Based on the results of multivariate Cox regression analysis, a sensitivity analysis was conducted by using the stepwise variable screening method. Model 1 excluded welders, Model 2 excluded frequent lunch breaks, and Model 3 excluded shift workers. All three models satisfied the PH assumption (Supplementary Table 1). The results of the sensitivity analysis showed that the effect of total years of work and exposure levels on lung-related disease was robust, indicating that higher concentrations of welding fume exposure were more likely to cause lung-related disease (Table 3).

## Discussion

In this retrospective cohort study, we investigated the exposure concentrations of welding fumes and metal ions in the air of workers in a vehicle factory in Wuhan and determined the exposure doses of different workers, as well as the levels of exposure. Second, a retrospective cohort study was conducted to explore the lung-related disease risk associated with welding fume exposure by determining the time from exposure to hazardous substances to the occurrence of lung-related diseases. Finally, through sensitivity analysis, the robustness of the model was verified. Different concentrations of welding fumes had different hazard ratios for lung-related diseases. In addition, the findings in our study may help us better understand the effects of welding fumes on workers' health.

Epidemiological studies have demonstrated that chronic exposure to welding fumes is associated with respiratory health effects, such as asthma, bronchitis, and lung function changes (16). However, due to the working environment, welding technology, and meteorological conditions, there are differences in individual morbidity and the course of disease, and the underlying pathogenesis is not completely clear. In recent years, it has been reported that welding fumes are closely related to systemic inflammation (14, 17). Welding fumes reduce the cytotoxicity of natural killer cell lymphokines, which activate killer cells (18). In addition, a retrospective study found a synergistic effect of smoking and welding fumes on lung cancer (19). Studies have shown that soluble Cr(VI) and Mn in welding fumes are associated with acute cellular and genotoxic effects *in vitro*, and insoluble ferric oxide has long-term effects on the human body and has potential lung cancer risk (20, 21). In this study, the concentration of welding fumes and the major metal ions Cr, Mn, Fe, and Ni were detected in the individual and fixed spot of the welders. The highest contact concentration of the welding fumes reached grade 4, which far exceeded the OEL limit, and the highest contact grades of Cr, Mn, Fe and Ni reached 3, 4 and 1, respectively. This study demonstrated that workers were exposed to different levels of welding fumes and metal ions, suggesting that plant and worker health should be monitored.

In addition, it was found that there were more males than females in the welder and non-welder groups *(p *<* 0.01)*. A study found that men were more likely than women to be exposed to noise, chemical hazards, and heavy physical labor (22). Compared with non-welders, welders had more nerve-related symptoms *(p *<* 0.05)* and lung-related symptoms *(p *<* 0.001)* in the last 3 months, and welders had significantly more lung-related diseases than non-welders *(p *<* 0.05)*. Welding fumes have been found to have a significant genetic effect on neurodegeneration (23), and there is a significant association between the metal Mn in welding fumes and migraine occurrence (24). Cr(VI) is a potent lung cancer carcinogen, and existing studies have found that exposure to welding fumes and Cr(VI) may cause squamous-cell carcinoma (25). This study found that welders were more inclined to squat and stand than non-welders *(p *<* 0.001)*, which was consistent with the actual situation of welders. In this study, welders were exposed to higher concentrations of welding fumes than non-welders, which is consistent with the results of the environmental investigation in this study. Welders were more likely than non-welders to wear dust masks *(p *<* 0.001)* and welding face screens *(p *<* 0.001)*, and the Abdel-Rasoul study found that effective mask wearing significantly improved the respiratory function of welders (26). The intense light and ultraviolet light produced during Almahmoud welding can damage the eyes, and the welding face screen and special goggles can effectively reduce damage to the eyes (27). In this study, it was found that the distribution of age and working life of non-welders was significantly different from that of welders, which was consistent with the actual situation in the factory. FEV1 was found to be lower in welders than in non-welders in the present study *(p *<* 0.047)*, which is consistent with the Ahmad study, where welders exposed to welding fumes had significantly decreased lung function (28).

Previous studies have shown that welders are more likely to develop lung-related diseases than non-welders (29, 30). Subgroup analysis showed that welders had a 2.17-fold increased risk of lung-related diseases compared with non-welders *(p *<* 0.01)*. In addition, the study found that male welders were 2.24 times more likely to develop lung-related diseases than non-welders *(p *<* 0.01)*, indicating that male welders are more likely to develop lung-related diseases and suggesting that male welders should pay more attention to personal protection. However, the incidence of lung-related diseases in welders was 1.97 times higher than that in non-welders *(p *<* 0.05)*, 2.84 times higher than that in non-welders *(p *<* 0.05)*, and 3.2 times higher than that in non-welders *(p *<* 0.01)*. Although few studies have reported on the effects of abdominal obesity and BMI on welders' lung-related diseases, studies have reported that fine particulate pollution in the air may aggravate the risk of respiratory disease in individuals with a high BMI (31). In addition, studies have found that welders develop at least two metabolic syndrome indicators and are more likely to develop obesity syndrome (32). Even among welders with a high level of education, the risk of developing lung-related diseases was 2.43 times higher than that among non-welders *(p *<* 0.01)*. Although studies have shown that welders with higher education have a higher awareness of individual protection and use of individual protection (33), welders have lower levels of exposure to welding fumes than non-welders and still have a high potential lung disease risk. The present study also revealed that welders who smoked were 2.44 times more likely to develop lung-related diseases than non-welders *(p *<* 0.01)*, and studies found that smoking welders exhibited significant inflammatory lung injury (34, 35). In addition, it was found that the incidence of lung-related diseases was 2.77 times higher in welders without lung-related symptoms than in non-welders in the last 3 months *(p *<* 0.001)*, which further revealed that the incidence of lung-related diseases was higher in welders without lung-related symptoms than in non-welders in the last 3 months *(p *<* 0.001)*; welders had a higher risk of developing lung-related diseases than non-welders.

In the univariate Cox proportional hazards model, total years of work was a protective factor for the occurrence of lung-related diseases, and the protective rate was 0.72, which may be due to the increase in working years and work experience. A more comprehensive understanding of process flow and hazard factors can allow individuals to take some protective measures against risk factors. This finding is consistent with the study that found that as workers work longer, they are much more likely to use protective equipment than those who do not have safety training (36). The risk ratio of lung-related diseases was 1.73. Among the people who took a nap regularly, 28.3% had lung-related symptoms in the past 3 months, and 16.9% of the total group had lung-related symptoms in the past 3 months. While napping can regulate sleep and increase one's energy level, welders who have developed symptoms of the disease and who are less energetic may be more prone to napping. The HR for lung-related disease was 5.66 in workers who worked three shifts compared to those who did not because shift work may have reversed the workers' biological clock, weakened the workers' immunity and made them more susceptible to lung-related diseases. In addition, high-high levels of exposure were more likely to cause lung-related disease than low-low levels of exposure. Studies have found that exposure to high concentrations of welding fumes may have a potential inflammatory effect on lung epithelial cells, and exposure to lower concentrations of welding fumes does not activate an inflammatory response in lung epithelial cells (14). Further combined factor analysis found that total years of work and exposure levels had an antagonistic effect on the occurrence of lung-related diseases, indicating that the accumulated experience of total years of work in different exposure levels may reduce the risk of lung-related disease from welding fume exposure.

Some limitations should be acknowledged in this study. First, we did not have a clear classification of welding fumes, which included hand-held arc welding and gas metal arc welding fumes, and were unable to identify the effects of different sources of welding fumes on lung-related diseases. For example, we have not measured the distribution of ultrafine particles in the lungs in different welding processes and can not make strict causal inference (37). In addition, the dispersion of PM2.5 and PM10 in the air from vehicle exhaust in cities also confounded the effects of lung-related diseases among workers (38). Second, this study identified lung disease that occurred in the subjects prior to the investigation through retrospective investigation. Although we repeatedly determined the time of disease onset of the subjects at the time of investigation, there may still be recall biases. In addition, the study was carried out in one factory, and it is difficult to generalize the results to other factories because of differences in manufacturing processes and plant construction times, and the recruitment of welders varies widely between factories.

## Conclusions

In conclusion, our study quantified the hazard ratios of exposure to different concentrations of welding fumes for the development of lung-related diseases in workers. Subgroup analyses quantified the risk ratios for lung-related disease among welders compared with non-welders in terms of different influencing factors. This paper may provide a reference for policy making to reduce the incidence of lung-related diseases in welders and further prevent the occurrence of lung-related diseases in welders. In view of the global marginalization of the prevention and control of occupational diseases, more surveys on the impact of welding fumes will be conducted in the future to provide evidence to support the development of occupational protection laws and the protection of the health of the occupational population.

## Data availability statement

The raw data supporting the conclusions of this article will be made available by the authors, without undue reservation.

## Ethics statement

The studies involving human participants were reviewed and approved by Ethics Committee of Wuhan Prevention and Treatment Center for Occupational Diseases: Wuhan Prevention and Treatment Center for Occupational Diseases, Jianghan Bei Lu 18, Wuhan, 430015, Hubei, PR China. The patients/participants provided their written informed consent to participate in this study.

## Author contributions

GL and XT designed the study. SW, YY, YLu, XC, HQ, WY, ZM, and GL collected the data. GL and JJ performed statistical analysis. GL, JJ, and YLi drafted this manuscript. XT and GY revised initial manuscript and had primary responsibility for final content. All authors were involved in the revisions and approved the final version of the manuscript.

## Funding

The China Association for Science and Technology in support topics related to post-epidemic recovery in Hubei (No. 20200608CG111306). Hubei Province Key Laboratory of open fund of Wuhan University of Science and Technology of Occupational Hazard Identification and Control (OHIC2018G07).

## Conflict of interest

The authors declare that the research was conducted in the absence of any commercial or financial relationships that could be construed as a potential conflict of interest.

## Publisher's note

All claims expressed in this article are solely those of the authors and do not necessarily represent those of their affiliated organizations, or those of the publisher, the editors and the reviewers. Any product that may be evaluated in this article, or claim that may be made by its manufacturer, is not guaranteed or endorsed by the publisher.
