# Supplementary material for: Risk for lung-related diseases associated with welding fumes in an occupational population: Evidence from a Cox model
Source: Front Public Health. 2022 Aug 25;10:990547. doi: 10.3389/fpubh.2022.990547 (PMC9455702; doi:10.3389/fpubh.2022.990547)
Supplement: Supplementary file 1 [file Table_1.docx]

Supplementary table 1 PH assumption of Multivariate cox model and sensitivity analysis model

|  |  | Chisq | Df | P |
| --- | --- | --- | --- | --- |
| Multivariate cox model | Welder | 0.216589 | 1 | 0.641651 |
|  | Total working years | 3.585304 | 1 | 0.058293 |
|  | Often take a nap | 0.641659 | 1 | 0.423111 |
|  | Whether to work shift | 2.062917 | 2 | 0.356487 |
|  | Exposure level | 4.030063 | 3 | 0.258236 |
|  | GLOBAL | 10.04565 | 8 | 0.261836 |
| Model1 | Total working years | 3.419778 | 1 | 0.06442 |
|  | Often take a nap | 0.694013 | 1 | 0.404803 |
|  | Whether to work shift | 2.014466 | 2 | 0.365228 |
|  | Exposure level | 3.931894 | 3 | 0.268913 |
|  | GLOBAL | 9.546889 | 7 | 0.215737 |
| Model2 | Welder | 0.163508 | 1 | 0.685947 |
|  | Total working years | 3.809654 | 1 | 0.050958 |
|  | Whether to work shift | 1.885155 | 2 | 0.389622 |
|  | Exposure level | 3.705055 | 3 | 0.295125 |
|  | GLOBAL | 9.41904 | 7 | 0.223954 |
| Model3 | Welder | 0.296908 | 1 | 0.585827 |
|  | Total working years | 3.751971 | 1 | 0.052745 |
|  | Often take a nap | 0.482044 | 1 | 0.487498 |
|  | Exposure level | 4.068713 | 3 | 0.254139 |
|  | GLOBAL | 8.233491 | 6 | 0.221491 |
